# Laparoscopic liver resection for a patient of hepatocellular carcinoma with von Willebrand disease: a case report

**DOI:** 10.1186/s40792-024-01960-4

**Published:** 2024-06-27

**Authors:** Oğuzhan Şal, Katsunori Sakamoto, Kei Tamura, Masahiko Honjo, Yusuke Nishi, Naotake Funamizu, Kohei Ogawa, Yasutsugu Takada

**Affiliations:** 1 Department of Hepato-Biliary-Pancreatic and Breast Surgery, Ehime University, Ehime University Graduate School of Medicine, 454 Kou, Shitsukawa, Toon, Ehime 791-0295 Japan; 2 Department of General Surgery, Istanbul University, Istanbul Faculty of Medicine, Istanbul, Turkey

**Keywords:** Hepatocellular carcinoma, Inherited coagulation disorder, Von Willebrand disease, Laparoscopic hepatectomy

## Abstract

**Background:**

The safety of laparoscopic hepatectomy for inherited coagulation disorders is unclear; however, the safety of open hepatectomy has been reported in several studies. Herein, we report the first case of a laparoscopic hepatectomy for a patient with von Willebrand Disease (VWD).

**Case presentation:**

A 76-year-old male with a history of chronic hepatitis C and VWD type 2B was advised surgical resection of a 4 cm hepatocellular carcinoma in segment 7 of the liver. The patient was diagnosed with VWD in his 40 s due to gastrointestinal bleeding caused by gastric erosion. The von Willebrand factor (VWF) ristocetin cofactor activity was 30%, and VWF large multimer deficiency and increased ristocetin-induced platelet agglutination were observed. The preoperative platelet count was reduced to 3.5 × 10^4^/μL; however, preoperative imaging findings had no evidence of liver cirrhosis, such as any collateral formations and splenomegaly. The indocyanine green retention rate at 15 min was 10%, and his Child–Pugh score was 5 (classification A). Perioperatively, VWF/factor VIII was administered in accordance with our institutional protocol. A laparoscopic partial hepatectomy of the right posterior segment was performed. The most bleeding during surgery occurred during the mobilization of the right lobe of the liver due to inflammatory adhesion between the retroperitoneum and the tumor. Bleeding during parenchymal transection was controlable. The duration of hepatic inflow occlusion was 65 min. The surgical duration was 349 min, and the estimated blood loss was 2150 ml. Four units of red blood cells and fresh frozen plasma were transfused at the initiation of parenchymal transection, and 10 units of platelets were transfused at the end of the parenchymal transection. On postoperative day 1, the transection surface drainage fluid became hemorrhagic, and emergency contrast-enhanced computed tomography showed extravasation in the greater omentum. Percutaneous transcatheter arterial embolization of the omental branch of the right gastroepiploic artery was performed. No further postoperative interventions were required. The patient was discharged on postoperative day 14.

**Conclusion:**

The indications for laparoscopic hepatectomy in patients with VWD should be carefully considered, and an open approach may still be the standard approach for patients with VWD.

## Introduction

Evidence on the safety profile of laparoscopic hepatectomy (LH) has been increasing [1]. However, the safety of LH in patients with inherited coagulation disorders (ICD), such as von Willebrand Disease (VWD), has not yet been demonstrated. VWD is the second most common ICD, with an estimated prevalence of 60.3 per million in high socioeconomic status countries [2]. Regarding the mechanism of the VWD, thrombocytes cannot adhere to the vessel walls due to von Willebrand factor (VWF) deficiency [3]. VWD has several subtypes, and type 2B VWD often presents with thrombocytopenia; however, the mechanism is unclear [4]. The main treatment for patients with VWD undergoing surgery is administering VWF/factor VIII concentrate or desmopressin [3, 4].

Hepatocellular carcinoma (HCC) is the most common primary liver malignancy and the third most common cause of cancer-related deaths among all human neoplasms worldwide [5]. Hepatotropic viral infections, such as those caused by hepatitis C viruses, are well-known causes of HCC [6]. The incidence of hepatotropic virus transmission has increased in patients with bleeding disorders owing to multiple transfusions [7]. Radical resection is the most effective treatment for HCC [6]. Recent advances in perioperative management, procedures, and instruments have enabled a safe increase in LH [6]. However, to our knowledge, no study has demonstrated the feasibility of LH in patients with VWD. Here, we present the case of a 76-year-old male with VWD who underwent LH for HCC located in segment 7 of the liver.

## Case presentation

A 76-year-old male with VWD was referred for HCC detected on abdominal computed tomography (CT) for routine follow-up of chronic hepatitis C. The patient had refractory nasal bleeding since childhood and a history of red blood cell transfusion. He was diagnosed with VWD type 2B by genetic analysis (point mutation in exon 28) in his 40 s due to gastrointestinal bleeding from gastric erosion. The von Willebrand factor (VWF) ristocetin cofactor activity, the plasma VWF antigen, and the plasma factor VIII activity were 30%, 97%, and 71%, respectively. VWF large multimer deficiency and increased ristocetin-induced platelet agglutination were observed. At the same time, he was diagnosed with chronic hepatitis C and was regularly followed up. When the patient was aged 68 years, he achieved a sustained virological response to direct-acting antiviral therapy.

**Fig. 1:**
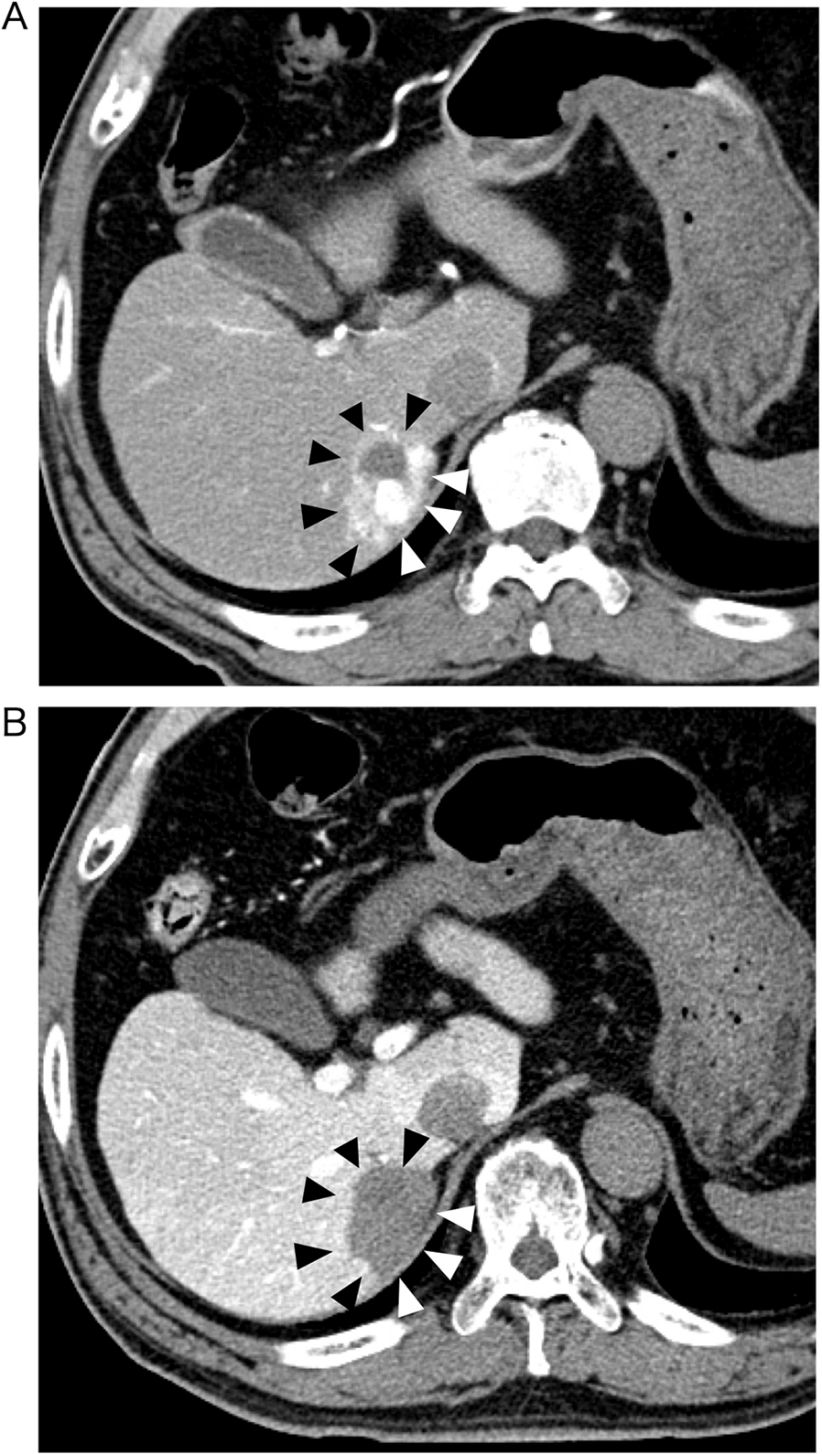
CT images with transcatheter angiography of the tumor. (Shown with arrow heads). A Arterial phase. The tumor shows early enhancement in a mosaic pattern. B Portal phase. Contrast is washed out in the portal phase

Preoperative CT showed a 4 cm diameter lesion in segment 7 of the liver, which was suitable for HCC. The tumor showed hyperenhancement in the arterial phase with portal venous washout on transcatheter angiography (Fig. 1), and the patient underwent transcatheter arterial chemoembolization (TACE) preoperatively. Before TACE, 2,000 U VWF/factor VIII concentrate (Confact F®) was administered. His preoperative platelet count was 3.5 × 10^4^/μL (Table 1), lasting from the diagnosis of VWD in his 40 s. Alpha-fetoprotein showed a normal level; however, des-γ-carboxy prothrombin level was increased to 112 mAU/mL. The indocyanine green retention rate at 15 min was 10%, and his Child–Pugh score was 5 (classification A). Preoperative imaging of the liver showed no evidence of portal hypertension, such as collateral formation or splenomegaly. No vascular invasion was detected on preoperative imaging; therefore, a laparoscopic partial hepatectomy of the right posterior segment was planned. Confact F^®^ was administered according to the protocol of the department of hematology at our hospital as follows: 4,000 U at 2 h before the operation; 2000 U at 2 h after the operation, 2000 U/d for the postoperative day (POD) 1–2; 1000 U/d for POD 3–4.

**Table 1:** Preoperative blood biochemistry data

Biochemical test			Complete blood count		
Albumin	4.0	g/dL	White blood cell count	4.6	× 10^3^/μL
Total-Bilirubin	0.8	mg/dL	Hemoglobin	14.3	g/dL
Aspartate aminotransferase	22	U/L	Platelet count	3.5	× 10^4^/μL
Alanine aminotransferase	16	U/L	Coagulation study		
Blood urea nitrogen	14	mg/dL			
Creatinine	0.86	mg/dL	Prothrombin activity	81.6	%
C-reactive protein	0.87	mg/dL	Prothrombin-international normalized ratio	1.00	
Indocyanine green retention rate at 15 min	10	%	Activated partial thromboplastin time	29.2	s
Tumor marker					
Alpha-fetoprotein	8	ng/mL			
Des-γ-carboxy prothrombin	112	mAU/mL			

**Fig. 2:**
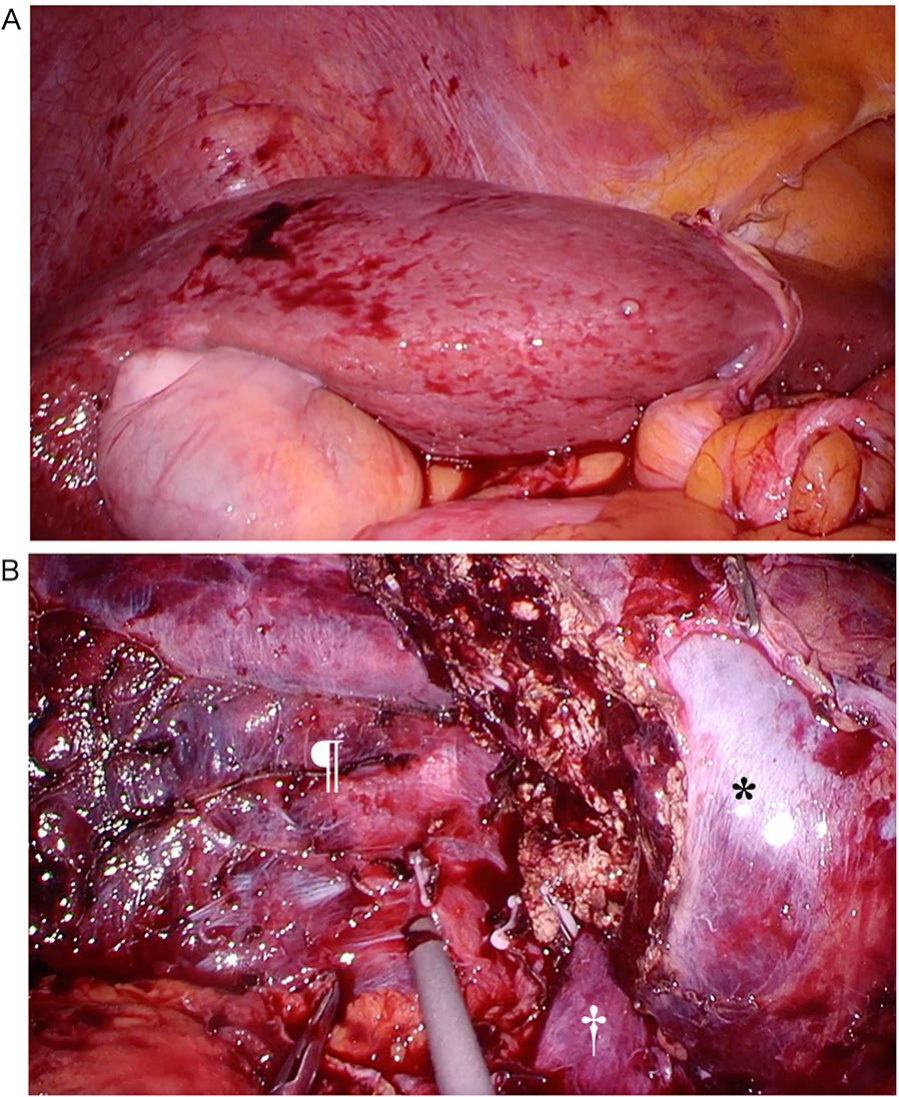
Laparoscopic findings. **A** The liver shows no cirrhotic changes; however, it shows chronic hepatitis. **B** After tumor resection. The bleeding was controlled. †, inferior vena cava; *, liver; ¶, diaphragm

**Fig. 3:**
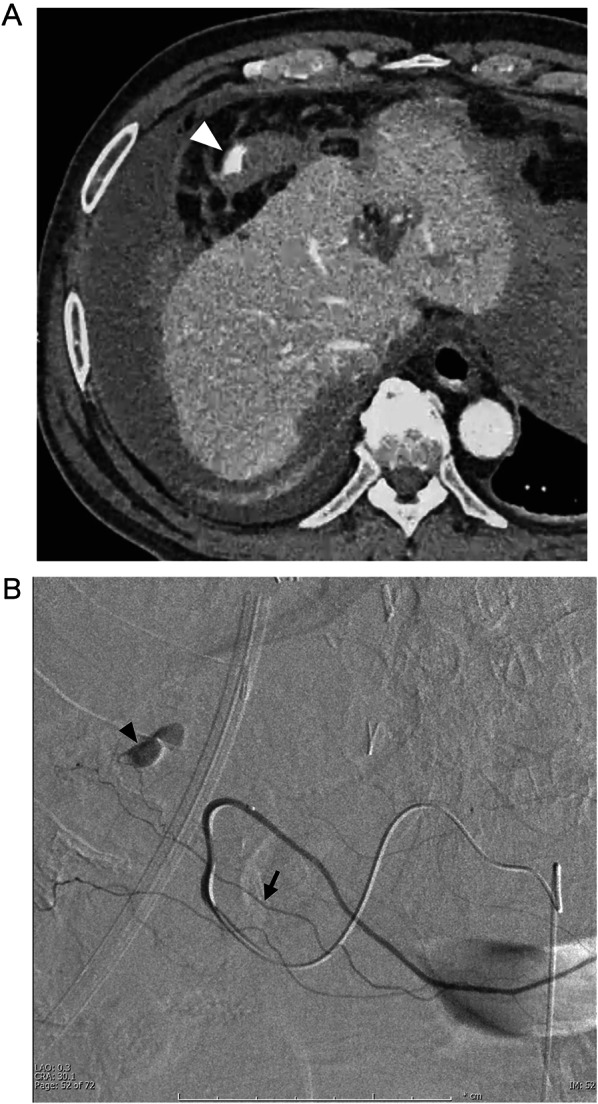
Radiological imaging of the postoperative day 1. **A** Contrast-enhanced CT. The arrowhead indicates extravasation from the greater omentum. **B** Emergency interventional radiology findings. The arrowhead indicates extravasation from the branch (arrow) of the right gastroepiploic artery. The branch was embolized peripherally, and the right gastroepiploic artery was preserved

**Fig. 4:**
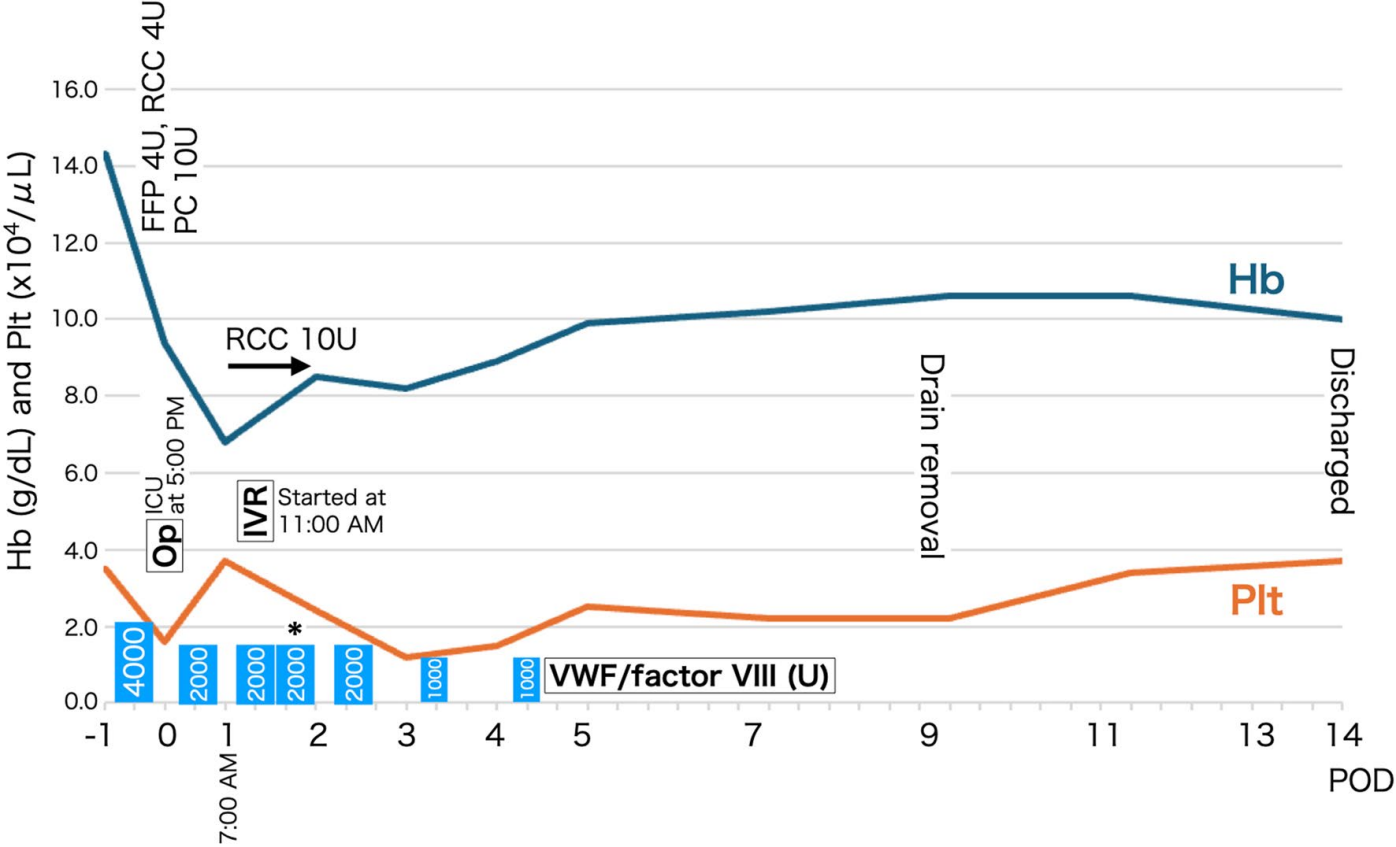
Clinical course of the patient. * Additional administration of von Willebrand factor/factor VIII concentrate (units). FFP, fresh frozen plasma; Hb, hemoglobin; IVR, interventional radiology; Op, operation; PC, platelet concentration; Plt, platelet count; POD, postoperative day; PT-INR, international normalized ratio of prothrombin; RCC, red blood cell concentration; U, unit; VWF/factor VIII, von Willebrand factor/factor VIII concentrate

**Fig. 5:**
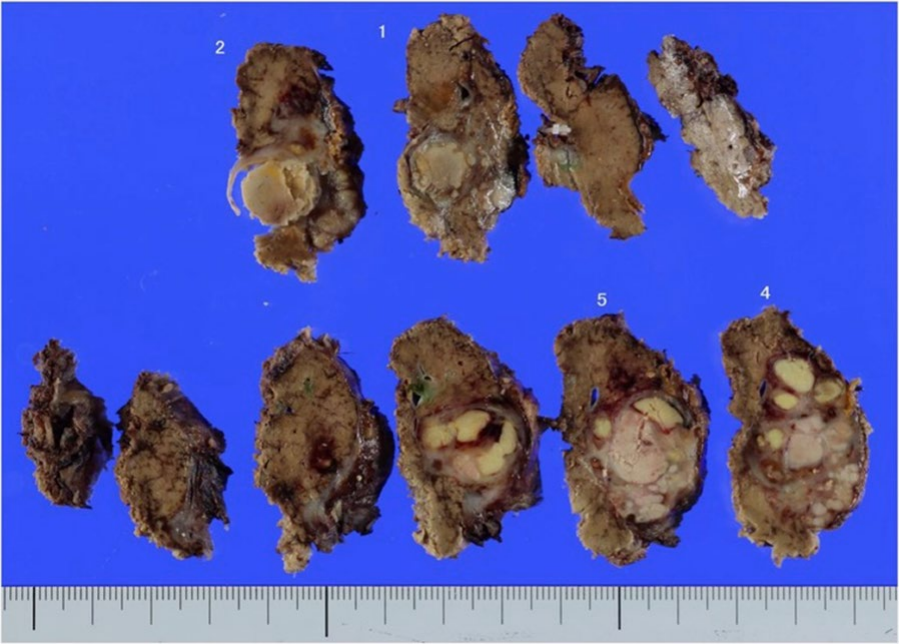
Specimen of the tumor. Pathological findings showed moderately differentiated hepatocellular carcinoma in a diameter of 37 mm. No vascular invasion was detected pathologically and the surgical margin was negative

The patient was placed in the reverse Trendelenburg position with a left tilt. Using the Hasson technique, a 12 mm trocar for the laparoscope was inserted into the umbilicus, and four trocars were placed beneath the right costal arch. A tourniquet was placed in the left hypochondrium to occlude the inflow of the liver hilum. Macroscopic findings of the liver revealed chronic hepatitis (Fig. 2A). The hepatic round ligament was transected, and the right liver was mobilized from the right diaphragm, retroperitoneum, and right adrenal gland to expose the inferior vena cava. LigaSureTM Maryland (Medtronics, Minneapolis, MN, USA) was the main energy device used to mobilize the right liver. Liver parenchyma was transected using the Cavitron Ultrasonic Surgical Aspirator (CUSA^®^) Excel system (Integra LifeSciences, Tullamore, Ireland) under ultrasonography guidance. The most bleeding during surgery occurred during the mobilization of the right lobe of the liver due to inflammatory adhesion between the retroperitoneum and the tumor. Bleeding during the parenchymal transection was under control (Fig. 2B). The duration of hepatic inflow occlusion was 65 min. A closed suction drainage tube was placed in the right subphrenic space to reach the surface of the transection. The operation lasted 349 min, and the estimated intraoperative blood loss was 2150 mL. Four units of red blood cells and fresh frozen plasma were transfused at the initiation of parenchymal transection and 10 units of platelets were transfused from the end of parenchymal transection. The surgery was completed with complete hemostasis, and the patient was transferred to the intensive care unit. On the morning of POD 1, the drain fluid became hemorrhagic and the hematocrit levels of whole blood decreased. Emergency contrast-enhanced CT revealed extravasation of the contrast inside the greater omentum on the caudal side of the liver. Percutaneous transcatheter arterial embolization of the omental branch of the right gastroepiploic artery was performed (Fig. 3). We added 2000 U of VWF/factor VIII concentrate after arterial embolization in addition to the planned protocol. No further postoperative interventions or transfusions were required. On POD 9, the drain was removed, and the patient was discharged on POD 14. The patient’s clinical course is shown in Fig. 4. Pathological examination of the specimen revealed a 37 mm mass suitable for HCC with a negative surgical margin (Fig. 5). Vascular invasion was not observed. The patient was doing well and had no recurrence at 5 months postoperatively.

## Discussion

VWD is a primary hemostasis deficiency presenting with mucosal bleeding, easy bruising, menorrhagia, and heavy bleeding during labor [8]. In the literature, several studies have reported about the safety and availability of conventional liver surgery in patients with ICD, including VWD [7, 9]. Furthermore, Denzer et al. reported that mini-laparoscopy-assisted liver biopsy was safe for patients with ICD [10]. None of the 61 patients had postintervention bleeding requiring reintervention [10]. Kobayashi et al. reported no significant difference in operative time, estimated blood loss, red blood cell transfusion rates, Clavien–Dindo classification 3–4 complications, or mortality rates between patients with and without ICD who underwent hepatectomy [7]. Yoshimoto-Haramura et al. reported that the postoperative complication rate did not increase in patients with ICD who underwent hepato-biliary-pancreatic surgery at qualified centers [9]. When limited to patients with VWD, several reports have described hepatectomies for patient safety [7, 12, 13]. Sato et al. reported a successful open hepatectomy in a patient with type 1 VWD patient [12]. However, their patient showed a preserved platelet count. Furthermore, type 2 VWD has been reported to cause more severe bleeding than type 1 [4]. Kobayashi et al. reported three successful cases of open hepatectomy for type 2 VWD; however, their cases had preserved platelet counts [7]. The clinical characteristics of the reported cases of VWD patients who underwent hepatectomy were presented in Table 2. In the present case, no preoperative findings suggested portal hypertension and thrombocytopenia was detected approximately 30 years ago. Therefore, the extremely low platelet count in the present case was considered to have been caused by type 2B VWD, which often coexists with thrombocytopenia [4]. Since the protocol of VWF/factor VIII administration in the present case was similar to that in previous reports [7, 12, 13], the extremely low preoperative platelet counts in the present patient might have caused the severe bleeding tendency.

**Table 2:** Clinical characteristics of the reported cases of the patients with von Willebrand disease who underwent hepatectomy

	Patients Age (year), sex	Approach	Procedure	Blood loss, mL	Red cell transfusion	Type	Primary disease, etiology	Preoperative treatment	Postoperative hospital stays, day	Postoperative complication	VWF ristocetin cofactor activity	VWF antigen	FVIII activity, %	APTT, sec	Platelet count, × 10^4^/μL	Child–Pugh classification	Administration of VWF/FVIII concentrate
Kokudo et al. [13] 2014	50, F	Open	Right hemi-hepatectomy	2100	Yes	2N	HCC, HCV	TACE	11	None	Normal	Normal	6	36.0	12.5	A	2 h before Op: 60U/kg 4 h after Op: 30U/kg 12 h after Op: 30U/kg 30U/kg for every 12 h until 7 dadys after Op
Kobayashi et al. [7] 2018	77, F	Open	Partial hepatectomy of S1	730 (110–2100)*^¶^	No	2A	HCC, HCV/HBV	None	13 (10–36)*^¶^	One Postoperative hemorrhage*^†^	No data	No data	6–8	27.9	16.1	A	
	70, M	Open	Left hemi-hepatectomy		No	2N	HCC, HCV	None			No data	No data		44.0	33.6	A	
Sato et al. [12] 2018	77, M	Open	Partial hepatectomy of S5 and S1	484	No	1	HCC, HCV	None	12	None	6	24	83	32.8	12.8	A	4800U in total
Present case	76, M	Lap	Partial hepatectomy of S7	2150	Yes	2B	HCC, HCV	TACE	14	Postoperative bleeding	30**	97**	71**	29.2	3.5	A	14,000U in total

*Of the 10 patients including 3 patients of VWD and 7 patients of haemophilia

**At the time of the diagnosis of VWD

^†^Treated by red cell transfusion (no intervention)

^¶^Inculding the data of the case of Kokudo et al.[13]

*APTT* activated partial thromboplastin time, *F* female, *FVIII* factor VIII, *HBV* hepatitis B virus, *HCC* hepatocellular carcinoma, *HCV* hepatitis C virus, *M* male, *Op* operation, *S* segment of the liver, *TACE* transcatheter arterial chemoembolization, *U* unit, *VWF* von Willebrand factor

In the current case, the most bleeding occurred at the retroperitoneum during mobilization of the right lobe due to inflammatory adhesions between the right liver and retroperitoneum, potentially caused by preoperative TACE. Careful consideration should be given to the indications for preoperative TACE of tumors located close to the surface of the liver. Although open conversion was considered intraoperatively, we considered that mobilization using a laparoscopic caudal view would have been more useful in the present case. Furthermore, open conversion may cause a greater amount of intraoperative bleeding from the abdominal wall and postoperative bleeding from the abdominal wall. Bleeding during parenchymal transection was not massive in the present case, which might be due to prior administration of frozen plasma during mobilization of the right liver. However, postoperative bleeding requiring intervention had occurred in the present case, and open conversion may have been a better approach.

The median intraoperative bleeding amount of LH (without other concomitant major operations) in our hospital from April 2016 to February 2024, when we standardized the LH procedure, was 100 mL (range, 0–2150 mL) in nonanatomical hepatectomies (*n* = 80) and left lateral sectionectomies (*n* = 9), and 200 mL (range, 0–1950 mL) in highly advanced anatomical hepatectomies (*n* = 23). The present case demonstrated the greatest intraoperative bleeding during LH at our hospital. Furthermore, we experienced no reoperation or interventional radiology due to postoperative bleeding in the LH, except in the present case. The above data are not an excuse for the clinical course of the present case; we concluded that a major part of the intra and postoperative bleeding in the present case might have been caused by the bleeding tendency of the patient. The patient in the present study also had refractory hematuria after transurethral biopsy for suspected prostate cancer 5 years before the hepatectomy, even with the appropriate administration of VWF/factor VIII. Furthermore, he experienced several episodes of gastrointestinal bleeding after polypectomy for colon polyps despite the appropriate administration of VWF/factor VIII.

In the present case, since omentum injury might have occurred outside the field of the laparoscopic view, more caution may be required for laparoscopic surgery. Notably, after abdominal exsufflation, untended bleeding can recur due to the discontinuation of pressure. Therefore, diligent hemostasis is a vital step in LH in patients with an ICD. Furthermore, the proper use of energy devices during surgery will prevent excessive bleeding and provide a better outcome. Nevertheless, postoperative bleeding may occur even with careful hemostasis in patients with ICD.

In conclusion, although we did not experience open conversion or reoperation, postoperative hemorrhage requiring percutaneous intervention occurred. Therefore, the indications for laparoscopic hepatectomy in patients with VWD should be carefully considered, and an open approach might still be standard for patients with VWD. Further studies are required to demonstrate the feasibility of LH in patients with VWD.

## Data Availability

All data that has been presented in this manuscript can be provided if necessary.

## Abbreviations

CT: Computed tomography
HCC: Hepatocellular carcinoma
ICD: Inheritance coagulation disorder
LH: Laparoscopic hepatectomy
POD: Postoperative day
TACE: Transcatheter arterial chemoembolization
VWD: Von Willebrand disease
VWF: Von Willebrand factor
